# 9-Meth­oxy-6a,11a-dimethyl-6a,11a-dihydro-6*H*-1-benzofuro[3,2-*c*]chromen-3-ol from *Dalbergia oliveri*

**DOI:** 10.1107/S1600536809034485

**Published:** 2009-09-09

**Authors:** Sujittra Deesamer, Warinthorn Chavasiri, Narongsak Chaichit, Nongnuj Muangsin, Udom Kokpol

**Affiliations:** a Department of Chemistry, Faculty of Science, Chulalongkorn University, Payathai, Bangkok 10330, Thailand; b Department of Physics, Faculty of Science and Technology, Thammasart University, Pathumthani 12121, Thailand

## Abstract

The title compound, commonly known as (+)-(6a*S*,11a*S*)-medicarpin, C_16_H_14_O_4_, was isolated from *Dalbergia oliveri* and displays a rigid mol­ecule consisting of four fused rings. The benzofuran system is inclined at an angle of 76.49 (2)° with respect to the chroman unit. The compound exists as a polymeric chain arising from inter­molecular O—H⋯O bonding.

## Related literature

For general background to (+)-(6a*S*,11a*S*)-medicarpin, see: Deesamer *et al.* (2007 ▶); Hargreaves *et al.* (1976 ▶). For a related structure, see: Aree *et al.* (2003 ▶).
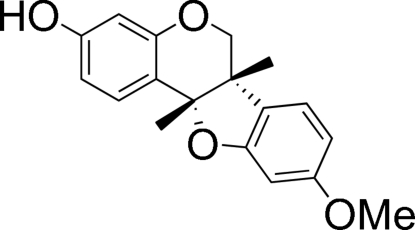


## Experimental

### 

#### Crystal data

C_16_H_14_O_4_
*M* *_r_* = 270.27
Monoclinic, 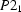

*a* = 6.6289 (3) Å
*b* = 8.7963 (4) Å
*c* = 11.3150 (5) Å
β = 99.4820 (10)°
*V* = 650.76 (5) Å^3^
*Z* = 2
Mo *K*α radiation
μ = 0.10 mm^−1^
*T* = 293 K
0.40 × 0.25 × 0.20 mm

#### Data collection

Bruker SMART diffractometer
Absorption correction: none
4783 measured reflections
1949 independent reflections
2867 reflections with *I* > 2σ(*I*)
*R*_int_ = 0.013

#### Refinement

*R*[*F* ^2^ > 2σ(*F* ^2^)] = 0.034
*wR*(*F* ^2^) = 0.093
*S* = 1.09
1949 reflections
182 parameters
1 restraint
H-atom parameters constrained
Δρ_max_ = 0.16 e Å^−3^
Δρ_min_ = −0.18 e Å^−3^

### 

Data collection: *SMART* (Bruker, 2006 ▶); cell refinement: *SAINT* (Bruker, 2006 ▶); data reduction: *SAINT*; program(s) used to solve structure: *SHELXS97* (Sheldrick, 2008 ▶); program(s) used to refine structure: *SHELXL97* (Sheldrick, 2008 ▶); molecular graphics: *ORTEP-3* (Farrugia, 1997 ▶); software used to prepare material for publication: *WinGX* (Farrugia, 1999 ▶).

## Supplementary Material

Crystal structure: contains datablocks global, I. DOI: 10.1107/S1600536809034485/ng2631sup1.cif

Structure factors: contains datablocks I. DOI: 10.1107/S1600536809034485/ng2631Isup2.hkl

Additional supplementary materials:  crystallographic information; 3D view; checkCIF report

## Figures and Tables

**Table 1:** Hydrogen-bond geometry (Å, °)

*D*—H⋯*A*	*D*—H	H⋯*A*	*D*⋯*A*	*D*—H⋯*A*
O4—H4*A*⋯O3^i^	0.82	2.07	2.882 (2)	169

Symmetry code: (i) 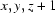.

**Fig. 1.**
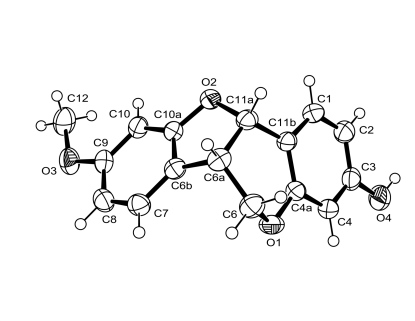
View of the title compound (50% probability displacement ellipsoids)

**Fig. 2.**
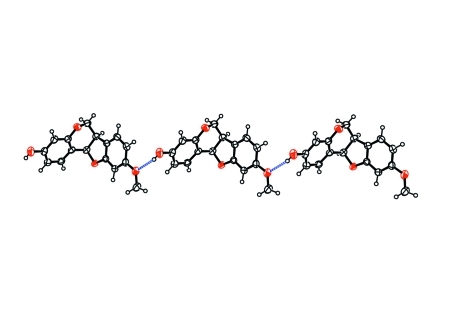
Packing diagram of a polymeric hydrogen bonding chain along the c axis.

## supplementary crystallographic information

### Comment

*Dalbergia Oliveri *Gamble is widely found in Thailand and used in traditional Thai medicine for treament of chronic ulcer. One of major compositions of CH_2_Cl_2_ crude products extracted from the heartwoods of *Dalbergia Oliveri * (Deesamer *et al.*, 2007) was (+)(6*aS*,11*aS*)-Medicarpin. It was identified as phytoalexin (Hargreaves *et al.*, 1976).

The rigid molecule of the title compound consists of four fused rings adopts a bent-shaped conformation. The benzofuran ring system is inclined at the angle of 76.49 (2)° with respect to the chroman moiety. The tetrahydropyranyl group adopts an envelope conformation with atom C6 deviates from the plane by 0.4144 Å.

The compound exists as a polymeric chain arising from intermolecular O—H···O bonding.

### Experimental

Four kilograms of dried and powder heartwoods of *D. oliveri* were extracted with hexane. The marc was then extracted with CH_2_Cl_2_, EtOAc and MeOH, respectively. The CH_2_Cl_2 _crudeextract was subjected to silica gel colume chromatography eluting with 60%EtOAc:Hexane to afford the title compound (3.92 g). The suitable single crystals of the title compound were recrystallized from acetone-water as colourless needle crystals.

m.p. 132.0–133.5°C; m/*z*: 270[*M*^+^]

The specific rotation of D3 as [α]_D_^+ ^223.1° (c 0.16 in acetone, at 20°C) indicated the absolute configuration to be (+)(6*aS*,11*aS*)-medicarpin.

^1^H-NMR (CDCl_3_): δ (p.p.m.) 3.55(1*H*,m,H-6a), 3.65 (1*H*, dd, J =10.9 and 10.9 Hz, H-6*ax*), 4.26 (1*H*, dd, J = 4.8, 10.9 Hz, H-6*eq*) and 5.23 (1*H*, d, J = 6.7 Hz, H-116*a*),

### Refinement

All non-hydrogen atoms were anisotropically refined. The hydrogen atoms were positioned geometrically and refined using a riding model, with C—H = 0.93Å (aromatic), 0.97Å (CH_2_) and 0.98Å (CH_3_), and O—H = 0.82 Å, and *U*_iso_(H) = 1.2*U*eq (C_aromatic_), 1.5*U*eq (C_CH2_), 1.5*U*eq (C_CH3_) and 1.2*U*eq (C_O_), respectively. In the structure, Friedel pairs [1949] were merged and the stereochemistry assumed from the specific rotation and the previously reported structure (Deesamer *et al.* 2007).

### Crystal data

**Table:**

C_16_H_14_O_4_	*Z* = 2
*M**_r_* = 270.27	*F*(000) = 284
Monoclinic, *P*2_1_	*D*_x_ = 1.379 Mg m^−^^3^
Hall symbol: P 2yb	Mo *K*α radiation, λ = 0.71073 Å
*a* = 6.6289 (3) Å	µ = 0.10 mm^−^^1^
*b* = 8.7963 (4) Å	*T* = 293 K
*c* = 11.3150 (5) Å	Needle, colourless
β = 99.482 (1)°	0.40 × 0.25 × 0.20 mm
*V* = 650.76 (5) Å^3^	

### Data collection

**Table:**

Bruker SMART diffractometer	*R*_int_ = 0.013
Radiation source: Mo	θ_max_ = 30.4°, θ_min_ = 1.8°
ω scans	*h* = −7→9
4783 measured reflections	*k* = −12→12
3198 independent reflections	*l* = −15→13
1949 reflections with *I* > 2σ(*I*)	

### Refinement

**Table:**

Refinement on *F*^2^	1 restraint
Least-squares matrix: full	H-atom parameters constrained
*R*[*F*^2^ > 2σ(*F*^2^)] = 0.034	*w* = 1/[σ^2^(*F*_o_^2^) + (0.0583*P*)^2^ + 0.0162*P*] where *P* = (*F*_o_^2^ + 2*F*_c_^2^)/3
*wR*(*F*^2^) = 0.093	(Δ/σ)_max_ < 0.001
*S* = 1.09	Δρ_max_ = 0.16 e Å^−^^3^
1949 reflections	Δρ_min_ = −0.18 e Å^−^^3^
182 parameters	

### Special details

**Table:**

Geometry. All e.s.d.'s (except the e.s.d. in the dihedral angle between two l.s. planes) are estimated using the full covariance matrix. The cell e.s.d.'s are taken into account individually in the estimation of e.s.d.'s in distances, angles and torsion angles; correlations between e.s.d.'s in cell parameters are only used when they are defined by crystal symmetry. An approximate (isotropic) treatment of cell e.s.d.'s is used for estimating e.s.d.'s involving l.s. planes.

### Fractional atomic coordinates and isotropic or equivalent isotropic displacement parameters (Å^2^)

**Table:**

	*x*	*y*	*z*	*U*_iso_*/*U*_eq_	
C1	0.0057 (2)	0.4390 (2)	0.69102 (16)	0.0414 (4)	
H1	−0.1252	0.398	0.6736	0.05*	
C2	0.0496 (3)	0.5393 (3)	0.78509 (16)	0.0458 (4)	
H2	−0.0512	0.5664	0.8294	0.055*	
C3	0.2455 (3)	0.6001 (2)	0.81356 (14)	0.0413 (4)	
C4	0.3960 (3)	0.5576 (2)	0.74813 (15)	0.0399 (4)	
H4	0.5278	0.5962	0.7678	0.048*	
C4A	0.3482 (2)	0.45666 (19)	0.65285 (14)	0.0351 (3)	
C6	0.4820 (3)	0.2767 (2)	0.53202 (17)	0.0426 (4)	
H6A	0.4918	0.1964	0.5915	0.051*	
H6B	0.5928	0.2632	0.4867	0.051*	
C6A	0.2800 (2)	0.26311 (19)	0.44829 (15)	0.0365 (3)	
H6A1	0.2669	0.1601	0.4149	0.044*	
C6B	0.2447 (2)	0.37674 (19)	0.34730 (14)	0.0338 (3)	
C7	0.3662 (3)	0.4270 (2)	0.26630 (15)	0.0393 (3)	
H7	0.5007	0.3937	0.2723	0.047*	
C8	0.2854 (3)	0.5275 (2)	0.17639 (16)	0.0424 (4)	
H8	0.367	0.5633	0.123	0.051*	
C9	0.0829 (3)	0.5752 (2)	0.16556 (14)	0.0388 (4)	
C10	−0.0419 (3)	0.5288 (2)	0.24674 (15)	0.0374 (3)	
H10	−0.1764	0.5621	0.2409	0.045*	
C10A	0.0455 (2)	0.43038 (19)	0.33679 (13)	0.0332 (3)	
C11A	0.0957 (2)	0.29610 (19)	0.51234 (15)	0.0363 (3)	
H11A	0.0382	0.2002	0.5358	0.044*	
C11B	0.1513 (2)	0.39649 (18)	0.62068 (14)	0.0343 (3)	
C12	−0.1997 (3)	0.7026 (3)	0.04448 (19)	0.0579 (5)	
H12A	−0.2266	0.7714	−0.0221	0.087*	
H12B	−0.2447	0.7474	0.113	0.087*	
H12C	−0.2719	0.6091	0.0244	0.087*	
O1	0.50439 (17)	0.42138 (16)	0.59175 (11)	0.0436 (3)	
O2	−0.05777 (17)	0.37602 (17)	0.42345 (10)	0.0401 (3)	
O3	0.0152 (2)	0.67276 (19)	0.07158 (11)	0.0518 (4)	
O4	0.2992 (2)	0.7012 (2)	0.90549 (12)	0.0550 (4)	
H4A	0.2102	0.7028	0.9479	0.083*	

### Atomic displacement parameters (Å^2^)

**Table:**

	*U*^11^	*U*^22^	*U*^33^	*U*^12^	*U*^13^	*U*^23^
C1	0.0296 (7)	0.0524 (10)	0.0410 (8)	−0.0031 (7)	0.0021 (6)	0.0049 (8)
C2	0.0390 (8)	0.0594 (11)	0.0391 (8)	0.0030 (8)	0.0070 (7)	0.0028 (8)
C3	0.0482 (9)	0.0424 (9)	0.0321 (7)	−0.0012 (7)	0.0026 (6)	0.0048 (7)
C4	0.0368 (7)	0.0448 (9)	0.0372 (7)	−0.0089 (6)	0.0034 (6)	0.0037 (7)
C4A	0.0323 (7)	0.0385 (8)	0.0343 (7)	−0.0031 (6)	0.0045 (6)	0.0059 (6)
C6	0.0361 (7)	0.0445 (10)	0.0460 (9)	0.0054 (7)	0.0030 (7)	0.0002 (7)
C6A	0.0372 (8)	0.0295 (7)	0.0414 (8)	0.0010 (6)	0.0022 (6)	−0.0007 (6)
C6B	0.0347 (7)	0.0306 (7)	0.0354 (7)	0.0000 (6)	0.0039 (6)	−0.0039 (6)
C7	0.0361 (7)	0.0411 (8)	0.0420 (8)	0.0012 (6)	0.0102 (6)	−0.0054 (7)
C8	0.0455 (9)	0.0459 (9)	0.0379 (8)	−0.0007 (7)	0.0130 (7)	−0.0007 (7)
C9	0.0474 (9)	0.0392 (9)	0.0296 (7)	0.0014 (7)	0.0055 (6)	−0.0027 (6)
C10	0.0359 (7)	0.0423 (8)	0.0331 (7)	0.0048 (6)	0.0035 (6)	−0.0023 (6)
C10A	0.0336 (7)	0.0359 (7)	0.0301 (6)	−0.0033 (6)	0.0049 (5)	−0.0032 (6)
C11A	0.0351 (7)	0.0338 (8)	0.0384 (8)	−0.0057 (6)	0.0010 (6)	0.0056 (6)
C11B	0.0310 (6)	0.0360 (8)	0.0342 (7)	−0.0033 (6)	0.0004 (5)	0.0071 (6)
C12	0.0621 (12)	0.0648 (13)	0.0431 (9)	0.0143 (11)	−0.0022 (9)	0.0089 (9)
O1	0.0305 (5)	0.0527 (8)	0.0482 (6)	−0.0086 (5)	0.0087 (4)	−0.0075 (6)
O2	0.0299 (5)	0.0536 (7)	0.0358 (5)	−0.0032 (5)	0.0020 (4)	0.0073 (5)
O3	0.0604 (8)	0.0588 (9)	0.0363 (6)	0.0084 (7)	0.0076 (5)	0.0106 (6)
O4	0.0639 (9)	0.0619 (9)	0.0394 (7)	−0.0071 (7)	0.0088 (6)	−0.0089 (6)

### Geometric parameters (Å, °)

**Table:**

C1—C2	1.376 (3)	C6B—C10A	1.389 (2)
C1—C11B	1.399 (2)	C7—C8	1.387 (3)
C1—H1	0.93	C7—H7	0.93
C2—C3	1.393 (3)	C8—C9	1.393 (2)
C2—H2	0.93	C8—H8	0.93
C3—O4	1.370 (2)	C9—O3	1.382 (2)
C3—C4	1.388 (3)	C9—C10	1.395 (2)
C4—C4A	1.392 (2)	C10—C10A	1.389 (2)
C4—H4	0.93	C10—H10	0.93
C4A—O1	1.372 (2)	C10A—O2	1.3709 (19)
C4A—C11B	1.400 (2)	C11A—O2	1.484 (2)
C6—O1	1.437 (2)	C11A—C11B	1.507 (2)
C6—C6A	1.512 (2)	C11A—H11A	0.98
C6—H6A	0.97	C12—O3	1.431 (3)
C6—H6B	0.97	C12—H12A	0.96
C6A—C6B	1.507 (2)	C12—H12B	0.96
C6A—C11A	1.547 (2)	C12—H12C	0.96
C6A—H6A1	0.98	O4—H4A	0.82
C6B—C7	1.389 (2)		
			
C2—C1—C11B	122.26 (15)	C6B—C7—H7	120.3
C2—C1—H1	118.9	C7—C8—C9	120.45 (16)
C11B—C1—H1	118.9	C7—C8—H8	119.8
C1—C2—C3	119.68 (17)	C9—C8—H8	119.8
C1—C2—H2	120.2	O3—C9—C8	116.15 (16)
C3—C2—H2	120.2	O3—C9—C10	122.38 (15)
O4—C3—C4	117.40 (16)	C8—C9—C10	121.46 (16)
O4—C3—C2	122.73 (17)	C10A—C10—C9	116.39 (15)
C4—C3—C2	119.87 (17)	C10A—C10—H10	121.8
C3—C4—C4A	119.56 (15)	C9—C10—H10	121.8
C3—C4—H4	120.2	O2—C10A—C6B	113.57 (14)
C4A—C4—H4	120.2	O2—C10A—C10	123.03 (14)
O1—C4A—C4	116.13 (14)	C6B—C10A—C10	123.39 (15)
O1—C4A—C11B	122.15 (14)	O2—C11A—C11B	108.81 (14)
C4—C4A—C11B	121.72 (14)	O2—C11A—C6A	106.09 (13)
O1—C6—C6A	112.14 (14)	C11B—C11A—C6A	112.62 (13)
O1—C6—H6A	109.2	O2—C11A—H11A	109.7
C6A—C6—H6A	109.2	C11B—C11A—H11A	109.7
O1—C6—H6B	109.2	C6A—C11A—H11A	109.7
C6A—C6—H6B	109.2	C1—C11B—C4A	116.87 (15)
H6A—C6—H6B	107.9	C1—C11B—C11A	121.33 (14)
C6B—C6A—C6	115.68 (14)	C4A—C11B—C11A	121.74 (14)
C6B—C6A—C11A	101.24 (12)	O3—C12—H12A	109.5
C6—C6A—C11A	112.20 (14)	O3—C12—H12B	109.5
C6B—C6A—H6A1	109.1	H12A—C12—H12B	109.5
C6—C6A—H6A1	109.1	O3—C12—H12C	109.5
C11A—C6A—H6A1	109.1	H12A—C12—H12C	109.5
C7—C6B—C10A	118.82 (15)	H12B—C12—H12C	109.5
C7—C6B—C6A	132.64 (14)	C4A—O1—C6	114.15 (13)
C10A—C6B—C6A	108.46 (13)	C10A—O2—C11A	106.43 (12)
C8—C7—C6B	119.42 (15)	C9—O3—C12	117.65 (15)
C8—C7—H7	120.3	C3—O4—H4A	109.5

### Hydrogen-bond geometry (Å, °)

**Table:**

*D*—H···*A*	*D*—H	H···*A*	*D*···*A*	*D*—H···*A*
O4—H4A···O3^i^	0.82	2.07	2.882 (2)	169

Symmetry codes: (i) *x*, *y*, *z*+1.
